# TbPIF5 Is a *Trypanosoma brucei* Mitochondrial DNA Helicase Involved in Processing of Minicircle Okazaki Fragments

**DOI:** 10.1371/journal.ppat.1000589

**Published:** 2009-09-25

**Authors:** Beiyu Liu, Jianyang Wang, Gokben Yildirir, Paul T. Englund

**Affiliations:** Department of Biological Chemistry, Johns Hopkins Medical School, Baltimore, Maryland, United States of America; Washington University School of Medicine, United States of America

## Abstract

*Trypanosoma brucei*'s mitochondrial genome, kinetoplast DNA (kDNA), is a giant network of catenated DNA rings. The network consists of a few thousand 1 kb minicircles and several dozen 23 kb maxicircles. Here we report that TbPIF5, one of *T. brucei*'s six mitochondrial proteins related to *Saccharomyces cerevisiae* mitochondrial DNA helicase ScPIF1, is involved in minicircle lagging strand synthesis. Like its yeast homolog, TbPIF5 is a 5′ to 3′ DNA helicase. Together with other enzymes thought to be involved in Okazaki fragment processing, TbPIF5 localizes in vivo to the antipodal sites flanking the kDNA. Minicircles in wild type cells replicate unidirectionally as theta-structures and are unusual in that Okazaki fragments are not joined until after the progeny minicircles have segregated. We now report that overexpression of *TbPIF5* causes premature removal of RNA primers and joining of Okazaki fragments on theta structures. Further elongation of the lagging strand is blocked, but the leading strand is completed and the minicircle progeny, one with a truncated H strand (ranging from 0.1 to 1 kb), are segregated. The minicircles with a truncated H strand electrophorese on an agarose gel as a smear. This replication defect is associated with kinetoplast shrinkage and eventual slowing of cell growth. We propose that TbPIF5 unwinds RNA primers after lagging strand synthesis, thus facilitating processing of Okazaki fragments.

## Author Summary

*Trypanosoma brucei* is a protozoan parasite that causes human sleeping sickness in sub-Saharan Africa. Trypanosomes are primitive eukaryotes and they have many unusual biological features. One prominent example is their mitochondrial genome, known as kinetoplast DNA or kDNA. kDNA, with a structure unique in nature, is a giant network of interlocked DNA rings known as minicircles and maxicircles. kDNA superficially resembles chain mail in medieval armor. The network structure dictates an extremely complex mechanism for replication, the process by which two progeny networks, each identical to their parent, are formed. These progeny networks then segregate into the daughter cells during cell division. One feature of this replication pathway, in which discontinuously synthesized strands of minicircles are joined together in a reaction involving an enzyme known as a helicase, is the subject of this paper. Since there is nothing resembling kDNA in human or animal cells, and since kDNA is required for viability of the parasite, enzymes involved in this pathway are promising targets for chemotherapy.

## Introduction

Trypanosomes and related parasites cause tropical diseases such as sleeping sickness and Chagas disease. As one of the earliest diverging eukaryotes that contain a mitochondrion [1], this group of parasites is well known for unusual biological properties. For example, their mitochondrial genome, known as kinetoplast DNA (kDNA), has an amazing and unprecedented structure, a giant DNA network residing in the cell's single mitochondrion [2],[3]. The network is a planar structure composed of interlocked DNA rings including several thousand minicircles and a few dozen maxicircles. Within the mitochondrial matrix the kDNA network is condensed into a disk-shaped structure that is positioned near the flagellar basal body, which resides in the cytoplasm. The kDNA disk, called the kinetoplast, is actually connected to the basal body by a transmembrane filament system named the tripartite attachment complex (TAC) [4].

Like mitochondrial DNA in other organisms, maxicircles encode ribosomal RNAs and a handful of mitochondrial proteins such as subunits of respiratory complexes. Many maxicircle transcripts require editing before they can serve as functional mRNAs. Editing is an unusual RNA processing reaction involving addition or deletion of uridylate residues at specific internal sites of mRNAs (reviewed in [5],[6]). In some transcripts, editing occurs on a massive scale, with uridylates introduced by editing constituting more than half of the sequence of the resulting mRNA. Minicircles encode small guide RNAs that serve as templates for editing, thereby controlling its specificity.

In this paragraph we will briefly discuss the kDNA replication mechanism in *T. brucei*, focusing on minicircles. The initial step in replication is the vectorial release of individual minicircles into the space, known as the kinetoflagellar zone (KFZ), between the kDNA disk and the membrane near the flagellar basal body [7]. Here the free minicircles encounter proteins that assemble and propagate a replication fork, resulting in unidirectional replication as theta structures. The progeny minicircles are thought to segregate in the KFZ, and then migrate to the antipodal sites, two protein assemblies that flank the kDNA disk and are positioned about 180° apart [8]. At this time the monomeric minicircle replication products contain either a single continuously synthesized leading strand or they contain unligated Okazaki fragments [9]. Within the antipodal sites the Okazaki fragments are processed. Although the detailed processing mechanism is unknown it probably involves several enzymes that localize within the antipodal sites. These enzymes, which have been studied to varying degrees, include structure-specific endonuclease I [10],[11], DNA polymerase β [12], and DNA ligase kβ [13]. These enzymes are thought to participate in removal of RNA primers and to fill and close the resulting gaps. The processed minicircles, containing either the newly synthesized leading strand or lagging strand and still containing at least one gap, are then attached to the network periphery by a topoisomerase II that is also situated in the antipodal sites [14],[15]. Since two minicircles are attached for every one removed, the network grows in size. Only when the minicircle copy number has doubled are their remaining gaps repaired, most likely by DNA polymerase β-PAK [16] and DNA ligase kα [13], two enzymes that reside within the kDNA disk. Then the network splits in two and its progeny, each identical to the parent, are pulled into the two daughter cells by their connection (via TAC) to the flagellar basal bodies [4].

Recently we discovered 8 proteins in *T. brucei* that are related to the *Saccharomyces cerevisiae* mitochondrial helicase ScPIF1, and we named them TbPIF1-8. Remarkably, six of these are localized at several different positions in the mitochondrion; of the other two, one is nuclear and the other appears to be in the cytoplasm [17]. We have so far studied only one of the mitochondrial proteins, TbPIF2, and have found it to be a helicase that is essential for maxicircle replication [17]. Here we report that TbPIF5 (Genbank accession No.: XP_847187; GeneDB accession No.: Tb927.8.3560) is a DNA helicase involved in minicircle Okazaki fragment processing, probably by unwinding the hybrid helices between RNA primers and the DNA template.

## Results

### Localization of TbPIF5

**Figure 1:**
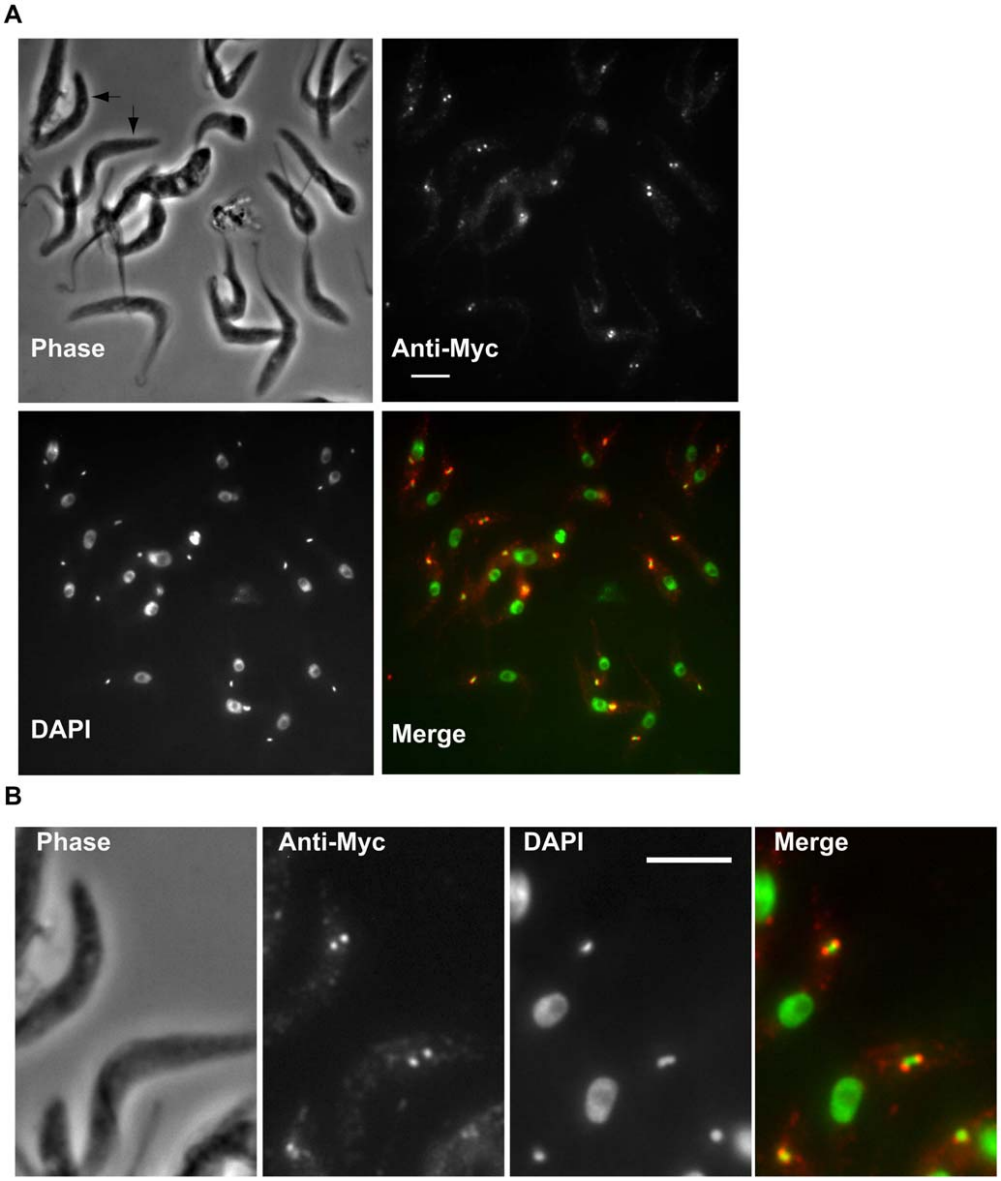
Localization of TbPIF5-Myc. Procyclic 927 cells harboring c-Myc-tagged TbPIF5 were fixed with 3% paraformaldehyde and then adhered to poly-L-lysine-treated slides. Immunostaining for TbPIF5-Myc used 1∶100 rabbit anti-Myc polyclonal antibody (Santa Cruz) and 1∶600 Alexa Fluor 568-conjugated goat anti-rabbit IgG (Molecular Probes). Conditions for fixing, permeabilizing, and staining cells were described [52]. In the merged image, anti-Myc is in red and DAPI in green. Arrows in panel A point out two cells with an enlarged magnification in panel B. Bar, 5 µm.

We previously localized TbPIF5 to the antipodal sites by expressing an ectopic gene encoding a TbPIF5-GFP fusion protein [17]. To localize TbPIF5 encoded at its endogenous locus, we introduced a sequence encoding a myc epitope at the 3′ end of one endogenous allele of *TbPIF5* gene. This protein would more likely be expressed at its normal level. Our immunofluorescence studies on this protein confirmed that TbPIF5 localizes within the antipodal sites (Fig. 1). Since almost all the cells in an asynchronous log phase culture had this localization, it is likely that this protein does not undergo significant change in its localization during the cell cycle.

### TbPIF5 is an ATP-dependent DNA helicase

**Figure 2:**
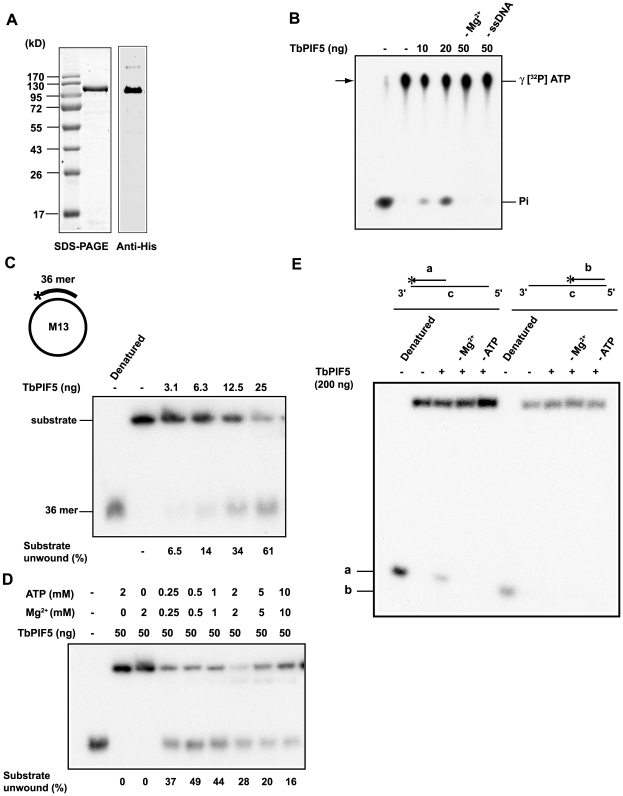
ATPase and helicase assays of recombinant TbPIF5. (A) Coomassie-stained SDS-PAGE gel and Western blot of purified recombinant TbPIF5. (B) Assay of TbPIF5 ATPase activity. The substrates and products were separated by polyethyleneimine thin layer chromatography; arrow shows origin. The [^32^P]Pi standard in the left-hand lane was prepared from [γ-^32^P] ATP by boiling 5 min in 1 M HCl. (C) Assay of TbPIF5 helicase activity. Substrates and products were separated by 12% polyacrylamide gel electrophoresis. (D) Helicase activity was measured at various concentrations of Mg^++^-ATP. (E) Assay of polarity of TbPIF5 helicase activity. Helicase substrates are diagrammed in Panels C (strand lengths are not to scale) and E (strand lengths for oligonucleotides a, b and c are 21, 21 and 90 nucleotides). * indicates 5′ ^32^P end label.

To determine whether TbPIF5 is actually a DNA helicase, we expressed it with a His-tag in *E. coli* and purified it by two steps of chromatography (Fig. 2A). Recombinant TbPIF5 hydrolyzes ATP in the presence of Mg^2+^ and M13 ssDNA (Fig. 2B), indicating that it has DNA-dependent ATPase activity. TbPIF5 also has helicase activity, releasing oligonucleotides that had been annealed to M13 single-stranded circles (Fig. 2C). As expected, Mg^2+^ and ATP are required for this reaction (Fig. 2D), and the optimal concentration for both was in the range of 0.5 or 1 mM (Fig. 2D). To determine the polarity of helicase activity, we constructed substrates (diagrammed in Fig. 2E) with a short oligonucleotide (either a or b; 5′ end-labeled with [^32^P]phosphate) annealed to either the 5′ or 3′ terminus of oligonucleotide c. Under conditions in which we observed dissociation of oligonucleotide a from the duplex structure, we could not detect dissociation of oligonucleotide b. Therefore, as predicted from its homology to the yeast mitochondrial helicase, we conclude that TbPIF5 has a 5′ to 3′ helicase activity (Fig. 2E).

### *TbPIF5* RNAi and knockout

To study the function of TbPIF5, we first tried RNAi using the pZJM vector [18]. Although ∼90% of the mRNA was degraded by 2 days after induction of RNAi (Inset, Fig. S1A), there was no effect on cell growth (Fig. S1A). Use of a stem-loop RNAi vector [18] gave the same result (data not shown). We then tried to knock out both alleles of *TbPIF5* by replacing each allele with a different drug marker. However, only one allele could be replaced as judged by Southern blot (Fig. S1B). Because knockout of both alleles may be lethal, we introduced into the cell an ectopic *TbPIF5* gene using the vector pLew79-MHTAP [19]. The ectopic gene expresses TbPIF5 only in the presence of tetracycline, and therefore it should allow deletion of the second genomic allele. For unknown reasons, this strategy was also unsuccessful using tetracycline concentrations ranging from 2–10 ng/ml (data not shown), and thus we failed to knock out both genomic alleles. As discussed in the following paragraph, we found unexpectedly that a higher level of tetracycline, which causes overexpression of *TbPIF5*, reduces the cell's growth rate.

### Overexpression of *TbPIF5* causes kDNA loss

**Figure 3:**
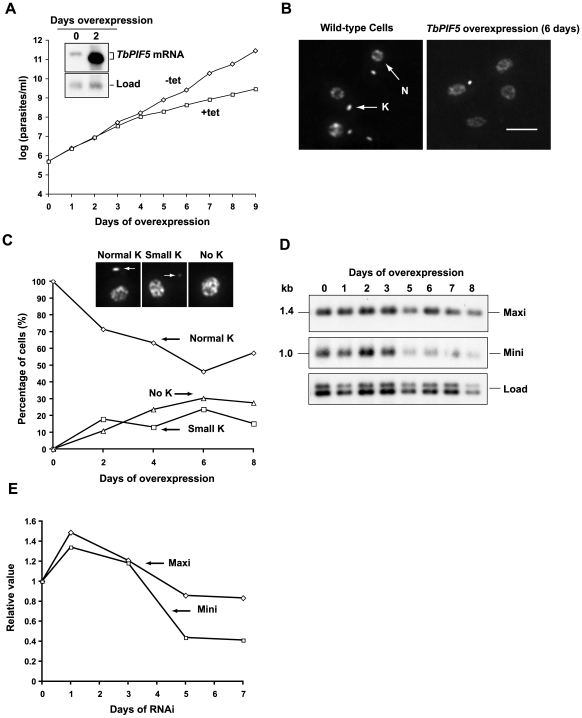
Effects of *TbPIF5* overexpression. (A) Effect of *TbPIF5* overexpression on cell growth. Overexpression was induced by addition of tetracycline (1 µg/ml) at day 0. The value of parasites/ml on the y-axis is the measured value times the dilution factor. Inset, Northern blot of mRNA level without or with overexpression. (B) Effect of overexpression on kinetoplast size as visualized by fluorescence microscopy of cells stained with DAPI (5 µg/ml). K, kinetoplast; N, nucleus. Bar, 5 µm. (C) Kinetics of kDNA loss as determined by visual analysis of images (>200 randomly-selected DAPI-stained cells for each time point). Inset images are examples of a cell with normal kinetoplast, small kinetoplast and no kinetoplast (kinetoplast is marked by arrow). (D) Effect of *TbPIF5* overexpression on minicircle and maxicircle abundance. Total maxicircles (Maxi) and minicircles (Mini) were detected by probing a Southern blot after the total DNA (10^6^ cell equivalents/lane) was digested with Hind III/XbaI and fractionated onto an agarose gel. The maxicircle probe detects only the 1.4 kb fragment, and only the 1 kb fragment derived from the heterogeneous minicircle population is shown. A hexose transporter fragment was probed as a loading control (Load). (E) Quantitation of the Southern blot in Fig. 3D showing maxicircle and minicircle species as indicated. Values represent the abundance of minicircle/maxicircle relative to its abundance in the uninduced cells. Values were normalized to load control.

Using the ectopic expression system discussed in the previous paragraph (except that both endogenous *TbPIF5* alleles were still present), we found that 2 days of treatment with 1 µg/ml tetracycline caused more than a 15-fold increase (judged by phosphorimaging) in *TbPIF5* mRNA (see northern blot inset in Fig. 3A). Furthermore, this treatment reduced the cell's growth rate 4 days after tetracycline addition (Fig. 3A), providing evidence that an elevated level of TbPIF5 is deleterious to the cell.

*TbPIF5* overexpression also caused shrinkage of kDNA networks as judged by DAPI staining of intact cells. Fig. 3B shows examples of fluorescence images of wild type cells and those that had undergone 6 days of overexpression. Fig. 3C shows kinetics of kDNA loss (determined by visual inspection of fluorescence images like those in panel B) following induction of overexpression. At day 6, only ∼50% of the cells had normal-sized kDNA, 20% had small kDNA, and 30% had no detectable kDNA. We then used a different approach to evaluate minicircle and maxicircle abundance following induction of *TbPIF5* overexpression. We digested total DNA with HindIII/XbaI, separated the fragments by agarose gel electrophoresis, and then probed a Southern blot for minicircles and maxicircles (Fig. 3D). After 5 days of overexpression, minicircle abundance decreased by more than half, while there was only a mild effect on the level of maxicircles (Fig. 3E). These results indicated that *TbPIF5* overexpression selectively affects minicircles.

**Figure 4:**
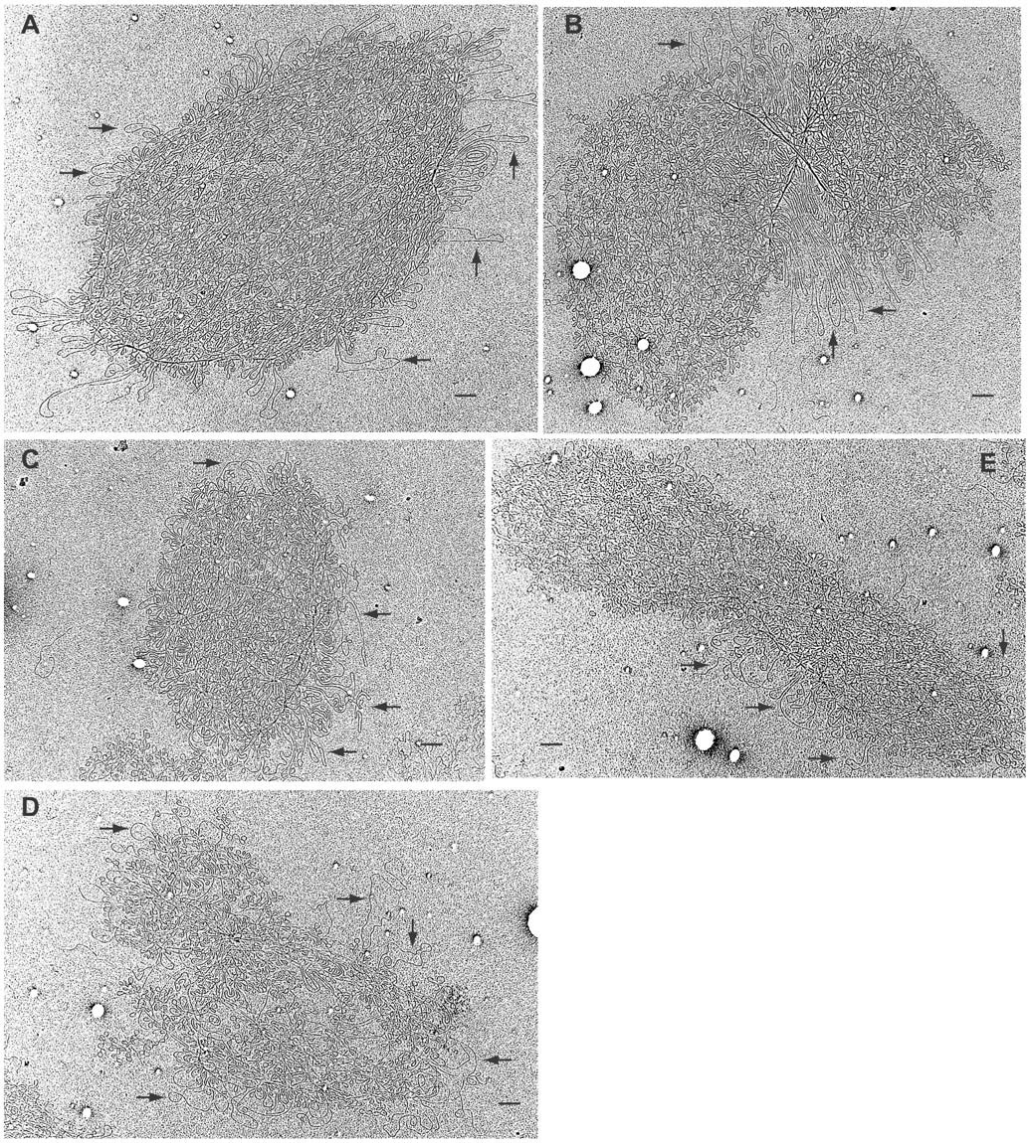
Electron micrographs of kDNA networks from *TbPIF5* overexpression cells. (A) and (B), kDNA isolated from wild-type cells. (C–E), kDNA isolated from *TbPIF5* overexpression cells six days after induction. Arrow, maxicircle loops. Bar, 500 nm.

We further examined the isolated kDNA networks by electron microscopy. The unit-sized network isolated from the uninduced cells has multiple maxicircle loops projecting from the periphery (arrows in Fig. 4A). In the late stage of replication, maxicircle loops usually concentrate in the central region between the two segregating daughter networks (arrows in Fig. 4B). After 6 days of *TbPIF5* overexpression, some networks have become smaller in size (Fig. 4C), and the structure of some networks is disorganized (Fig. 4D). As usual, we observed multiple maxicircle loops extending from the edge of the networks in different stage of replication. However, they do not always concentrate in the central region of the double-sized network that is undergoing segregation (see an example in Fig. 4E).

### Free minicircle analysis

**Figure 5:**
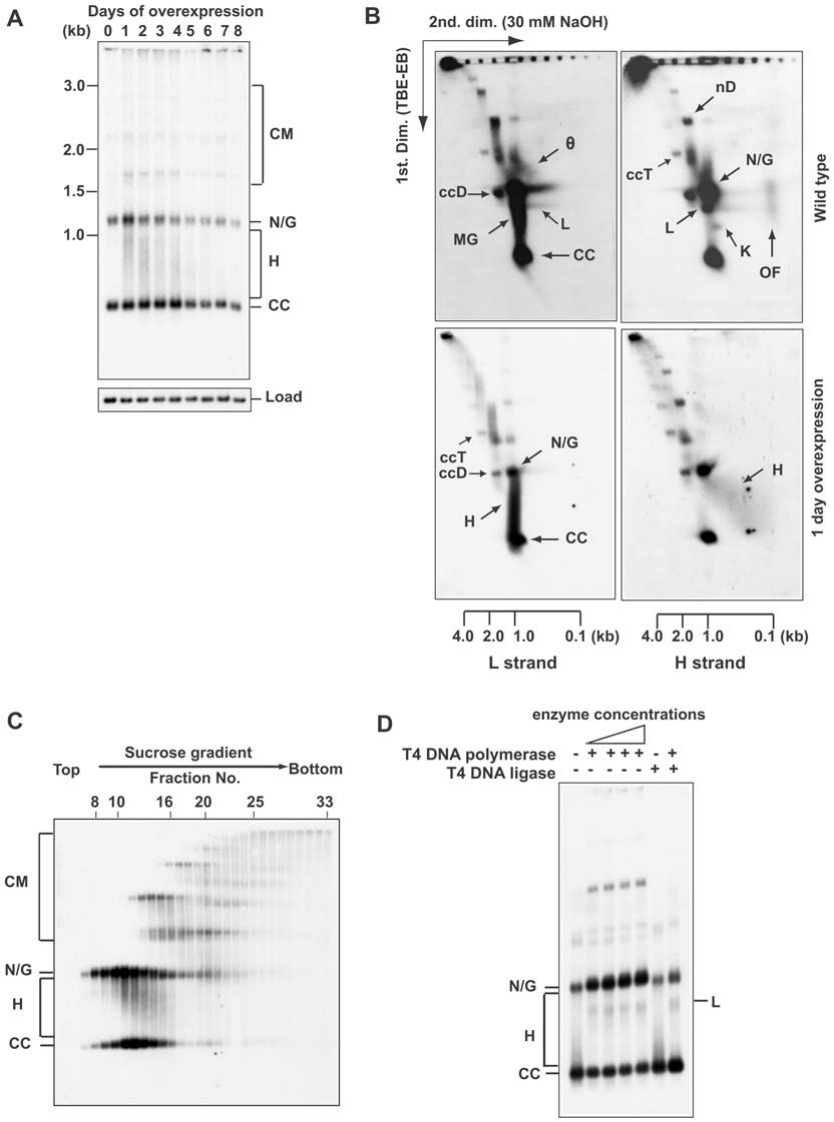
Effect of *TbPIF5* overexpression on free minicircle intermediates. (A) Total DNA (10^6^ cell equivalents/lane) was fractionated on a 1.5% agarose gel in TBE buffer (both the gel and running buffer contained 1 µg/ml ethidium bromide). A southern blot was probed for minicircles and hexose transporter (Load). (B) Neutral/alkaline two-dimensional gel electrophoresis. Total DNA from 3×10^7^ wild type or induced cells (1 day overexpression) was fractionated on a two-dimensional gel. Strand-specific hybridizations were conducted with synthetic oligonucleotide probes. The upper panel shows a longer exposure version of the same 2-D gel used in Fig. 3F of our previous paper [20]. The scales below the panels indicate the sizes of linear markers in the second dimension. (C) Sedimentation of free minicircle intermediates in a 5–20% sucrose gradient. Fractions were collected from the top (1 ml fractions), subjected to electrophoresis, and assayed by probing a Southern blot [20]. (D) Gel electrophoresis (using conditions described for Panel A) of free minicircles treated with various enzymes. Total free minicircles were purified on the sucrose gradient in Panel C by pooling fractions 8 to 16 and ethanol precipitating the DNA. The free minicircles were then treated with T4 DNA polymerase (0.6 U, 1 U, 2 U and 3 U, New England Biolabs) and/or T4 DNA ligase (400 U, New England Biolabs). CM, catenated minicircles; N/G, nicked/gapped minicircles; CC, covalently-closed minicircles; θ, theta-structure; k, knotted minicircle; H, fraction H; ccD, covalently-closed dimer; ccT, covalently-closed trimer; nD, nicked dimer; L, linearized minicircle; MG, multiply-gapped minicircle; OF, Okazaki fragments.

To investigate whether minicircle loss caused by *TbPIF5* overexpression is due to an effect on replication, we fractionated total DNA on an agarose gel in the presence of ethidium bromide and detected free minicircle replication intermediates by probing a Southern blot (Fig. 5A). After 5 days of overexpression, both covalently-closed and gapped/nicked minicircles decreased by about half, consistent with the decrease in total minicircle abundance. One unexpected consequence of *TbPIF5* overexpression was the appearance of a heterogeneous population of minicircle species migrating as a smear between covalently-closed and gapped/nicked minicircles. This smear, never before observed and which we call fraction H, is most prominent on days 1 to 4.

### Characterization of fraction H

We next fractionated total DNA using neutral/alkaline 2-dimensional gel electrophoresis and analyzed free minicircle species by strand-specific hybridization (Fig. 5B). In the first dimension, minicircle species were separated in TBE buffer containing ethidium bromide (conditions identical to those used for the gel in Fig. 5A). In the second dimension, run in 30 mM NaOH, the double-stranded DNA was denatured. Using 5′-^32^P -labeled synthetic oligonucleotides, we separately probed for L- (the leading strand) and H-strands (the lagging strand). The probes were complementary to sequences near the 5′ end of the L-strand and within the first Okazaki fragment on the H-strand. Interpretation of these gels was aided by comparison with our previous 2-D gels of minicircles from the closely-related parasite *T. equiperdum* [9] as well as from *T. brucei* [20]. As mentioned in the Introduction, these and other studies had shown that minicircles replicate unidirectionally via theta structures with the L-strand synthesized continuously and the H-strand discontinuously with ∼100 nucleotide Okazaki fragments. This mechanism is unusual in that Okazaki fragments are not joined until the θ-structures had segregated into monomeric products; joining is thought to occur within the antipodal sites [9].

In the control 2-D gel of wild type free minicircle intermediates (Fig. 5B, upper panels), we show a fairly long exposure to reveal the unjoined Okazaki fragments (OF) derived from multiply-gapped circles (MG) and the diagonal of growing L-strands ranging in size up to ∼1 kb derived from θ-structures (θ). Joining of most of the Okazaki fragments in a minicircle converts multiply-gapped minicircles to nicked or gapped minicircles (N/G). Some minor minicircle species previously identified in wild type *T. equiperdum* and *T. brucei* such as the knotted minicircle (K), linearized minicircle (L), nicked dimer (nD), and covalently-closed dimer (ccD) are not relevant to this paper and not discussed here [9],[20],[21].

Two-dimensional gels of minicircles from cells undergoing *TbPIF5* overexpression for 1 day (Fig. 5B, lower panels) differed markedly from those from wild type cells (Fig. 5B, upper panels). We found that fraction H has a ∼1 kb L-strand template and in the next paragraph we will present strong evidence that this strand is circular. These L-strands form a smear extending from CC to N/G (H, left lower panel in Fig. 5B). The reason for smearing is that prior to denaturation they had been paired with H strands varying in size. The latter molecules form a diagonal, never observed previously, in the size range of 0.1 to near 1 kb (H, right lower panel in Fig. 5B). Thus, fraction H likely consists of a circular L-strand paired with a family of growing H strands. Since the probe detects only the first Okazaki fragment to be synthesized, the H-strand fragments in the diagonal must include the first and form a family of ligated contiguous Okazaki fragments. Strand-specific hybridization also suggested a decrease in level of growing L-strands on θ-structures (compare L-strand diagonal, designated θ, in left upper panel in Fig. 5B with corresponding area of left lower panel), although since different exposures were used it is not possible to make a firm conclusion on this point.

To further characterize fraction H, we purified free minicircles by sucrose gradient centrifugation (Fig. 5C) and treated these molecules with T4 DNA polymerase (plus all four dNTPs), T4 DNA ligase (plus ATP), or both together (Fig. 5D). DNA polymerase alone converts fraction H to the position of gapped/nicked minicircles, but DNA ligase alone barely affects the mobility of fraction H. However, both enzymes together convert a substantial portion of fraction H to covalently-closed minicircles. This experiment not only indicates that fraction H is a gapped molecule with ligated Okazaki fragments but also provides evidence that the L-strand of fraction H is a circle.

### Examination of minicircle primers

**Figure 6:**
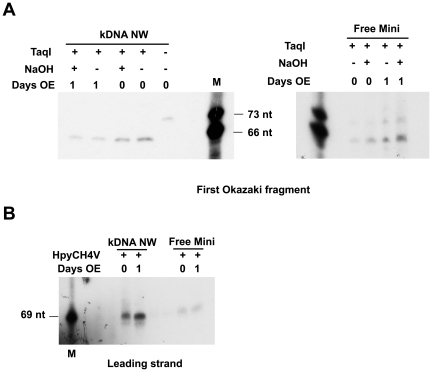
Effect of *TbPIF5* overexpression on replication primers. See [11] for experimental details of this experiment. (A) Analysis of 5′ ribonucleotides on the first Okazaki fragment. kDNA networks and free minicircle intermediates were isolated from cells without *TbPIF5* overexpression or after overexpression for 1 day. DNA was digested with TaqI and treated with 0.3 M NaOH as indicated; alkali treatment would remove ribonucleotides and alter fragment mobility. After fractionation on a denaturing 9% polyacrylamide gel, a Southern blot was probed for the first Okazaki fragment with a ^32^P-labeled oligonucleotide. (B) Analysis of primers on the leading strand. kDNA networks and free minicircle intermediates were digested with HpyCH4V, and after electrophoresis a Southern blot was probed with a ^32^P-labeled oligonucleotide complementary to the 5′ end of the leading strand. M, size marker; OE, overexpression.

If TbPIF5 is involved in primer removal, it is possible that its overexpression might reduce the number or length of primers on either free minicircles or those linked to the network. We previously reported that in *T. brucei* there are no ribonucleotides on the 5′ end of either the newly synthesized L-strand or the first Okazaki fragment on minicircles that were linked to the network [11]. However, we never had searched for primers on free minicircles. Using the strategy we developed previously [11], we investigated whether primers were present before and after *TbPIF5* overexpression (Fig. 6). We isolated kDNA networks and free minicircle intermediates (from both uninduced and 1 day overexpression cells), digested them with TaqI, and fractionated the products on a denaturing 9% polyacrylamide gel. We then probed a Southern blot for the first Okazaki fragment. This fragment, containing ∼73 nucleotides but with a slightly heterogeneous 3′ end, had been converted by TaqI to a slightly smaller fragment (66 nucleotides) with a homogeneous 3′ end (Fig. 6A). This species, whether derived from free minicircles or network minicircles, was not altered by alkali treatment, indicating that there are no ribonucleotides on its 5′ end or anywhere else within the molecule. Using a similar strategy, we searched for ribonucleotides at the 5′ terminus of the continuously-synthesized L-strand. We cleaved the minicircles with HpyCH4V, which release a 69 nucleotide terminal L-strand fragment (Fig. 6B). Again, there is no ribonucleotide attached at the 5′ end of the newly-synthesized L-strand.

## Discussion

In a recent search for *T. brucei* mitochondrial DNA helicases, we found that the genome encodes 8 proteins related to ScPIF1, a mitochondrial helicase of *S. cerevisiae*. Remarkably, 6 of the *T. brucei* PIF1-related gene products are mitochondrial [17]. Here we report the properties of one of these enzymes, TbPIF5, which is localized in the antipodal sites (Fig. 1). As shown in Fig. 2, we found that a recombinant protein had helicase activity, with a 5′ to 3′ polarity, similar to that of the yeast homolog [22]. We did not observe a phenotype following RNAi of *TbPIF5*, even though ∼90% of the mRNA was depleted within 2 days (Fig. S1). We could knock out one, but not both alleles of *TbPIF5*, raising the possibility that the gene is essential. Surprisingly, the genome of a related kinetoplastid, *Leishmania major*, encodes only 7 PIF1-like helicase genes, and the counterpart of *TbPIF5* gene is apparently absent [17]. Although this fact might support an argument that *TbPIF5* could be dispensable we cannot rule out the possibility that other *PIFs* may take over *TbPIF5*'s functions in *L. major*. Like *T. brucei*, the *T. cruzi* genome contains 8 genes related to *ScPIF1*.

Although RNAi and single allele knockouts did not affect cell growth or kDNA size as determined by DAPI staining, we did observe a striking effect of *TbPIF5* overexpression on the replication of minicircles. Not only was there a slowing of growth and loss of kDNA minicircles (Fig. 3), but there was an alteration in joining of Okazaki fragments (Fig. 5). Before we discuss these new data, we will review what is known about primer removal and other processing reactions of minicircle Okazaki fragments. We will also review Okazaki fragment joining in the nucleus of other eukaryotes.

There is a fundamental difference between processing of trypanosome minicircle Okazaki fragments with that in other cells. In either prokaryotes or eukaryotes, Okazaki fragment primers are generally removed and fragments are ligated immediately after their synthesis [23]. In trypanosome mitochondria, on the other hand, minicircle Okazaki fragments are not joined until after the progeny minicircles have segregated. In *T. brucei*, theta-type replication apparently occurs in the KFZ, and then the segregated progeny are thought to migrate to the antipodal sites (probably with one sister minicircle going to each antipodal site [2]). At this stage the progeny molecules with a newly-synthesized H-strand are designated multiply-gapped circles, and the gaps are positioned between the ∼100 nucleotide Okazaki fragments [9],[24]. The presence in the antipodal sites of multiply-gapped minicircles (with a 3′ OH terminus on each Okazaki fragment) explains the intense *in situ* labeling of these sites by terminal deoxynucleotidyl transferase and a fluorescent dNTP [25]. The antipodal sites also contain enzymes that likely function in primer removal and gap repair. These include structure-specific endonuclease I (SSE-1, homologous to the 5′ exonuclease domain of bacterial DNA polymerase I) [26]. RNAi of *SSE-1* confirms its involvement in primer removal [11]. It is likely that following primer removal all but one of the gaps are repaired by DNA polymerase β and DNA ligase kβ, both of which are positioned in the antipodal sites [13],[16]. Following repair of most but not all gaps, these minicircles, together with their sister minicircles (also containing a single gap adjacent to or overlapping the L strand start site) are reattached to the network periphery by a topoisomerase II that is also positioned in the antipodal sites [14],[15]. Neither free minicircles nor network minicircles from procyclic *T. brucei* contain 5′ ribonucleotides derived from primers (Fig. 6 and [11]), suggesting that in these cells primer removal is efficient. In contrast, we found one or two ribonucleotides on network minicircles (both on the leading strand and at least on the first Okazaki fragment), in cells that had undergone RNAi knockdown of SSE-1 [11]. However, the newly-synthesized L-strands on network minicircles in *T. equiperdum* bloodstream forms have one or two 5′ ribonucleotides [27] and in the related parasite *C. fasiculata* has up to six [28]. No residual RNA primer was found associated with the minicircle H strand fragments in *T. equiperdum* [29]. Finally, we do not know for any of these parasites the initial length of the primer or all of the enzymes involved in their removal. *C. fasciculata* has a mitochondrial RNase H1 [30] and a comparable enzyme is found in *T. brucei* [31]; this enzyme may also contribute to primer removal.

To understand processing of minicircle Okazaki fragments it is essential to consider the enzymology of this complex pathway in nuclei of other eukaryotes. Proteins involved in this process include flap endonuclease 1 (FEN1), RNase H, Dna2p, replication protein A (RPA), DNA polymerase δ, and DNA ligase I [23]. RNase H removes the primer one nucleotide upstream of RNA-DNA junction [32], and the remaining ribonucleotide is then cleaved by FEN1 [33]. *S. cerevisiae* also contains an RNase H-independent pathway in which DNA polymerase δ can strand-displace the RNA primer, forming a flap intermediate. Most flap intermediates are short and can be cleaved by FEN1 itself [34]–[37]. However, long flaps (>30 bases) may also be generated by DNA polymerase δ. The long flap is then coated by the single-strand binding protein RPA, which recruits Dna2p, a protein with both 5′ to 3′ helicase and nuclease activities. Dna2p cleaves the long flap into a shorter flap that is subsequently removed by FEN1. Finally the resulting gap is repaired by polymerase δ and ligase I [38],[39]. Recent studies in yeast have uncovered a role for PIF1 helicase in these reactions (ScPIF1 is found in both the mitochondria and the nucleus) [40]–[42]. The genetic interaction between *PIF1*, *DNA2* and a subunit of pol δ (*POL32*), together with the biochemical studies [43],[44], indicate that Pif1p may assist pol δ in generating the flap, which is processed subsequently by Dna2p [45]. The mechanism by which Pif1p functions in this process is still unclear.

**Figure 7:**
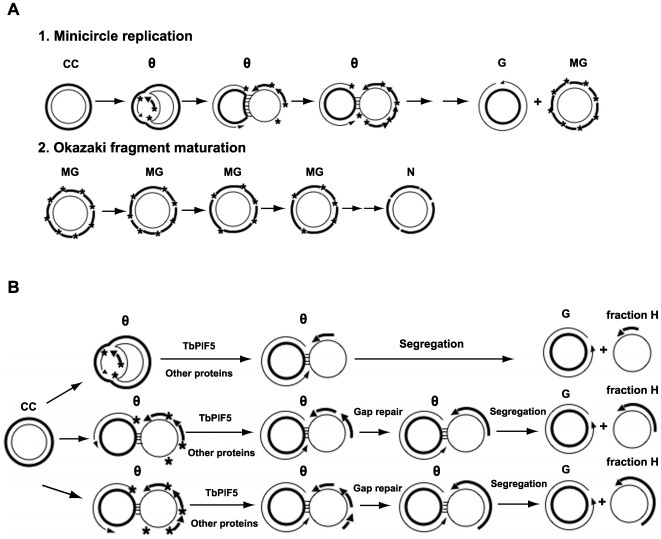
Comparison of the normal free minicircle replication mechanism with that in cells overexpressing *TbPIF5*. (A) Replication scheme showing (line 1) conversion, via theta structures (θ), of covalently-closed parental minicircles to gapped (G) and multiply-gapped (MG) progeny. The MG molecules are then converted (line 2) to nicked minicircles (N). The thick strand is H, which is synthesized discontinuously by Okazaki fragments, and the thin strand is L, which is synthesized continuously. * is an RNA primer and horizontal lines linking two circles in the θ-structures represent base pairs in the unreplicated portion. (B) When TbPIF5 is overexpressed, it binds to θ-structures and triggers primer removal. Removal of primers between the newly-synthesized Okazaki fragments generates gaps which can then be repaired. A similar removal of primers that have not yet been used for initiation blocks subsequent lagging strand replication. Leading strand synthesis is essentially unaffected and proceeds to completion allowing segregation. This process generates fraction H, a family of minicircles with a circular parental L-strand and increasing numbers of joined Okazaki fragments (ranging in size from 73 nt, the first Okazaki fragment, to 1 kb). This diagram shows the generation of three different species of fraction H, in which one, two or three Okazaki fragments are synthesized and then subjected to primer removal and joining. For simplicity, this diagram was not drawn to scale. We speculate that reactions in line 1 of panel A occur in the KFZ and those in line 2 take place in the antipodal sites. We further speculate that all reactions in Panel B occur in the KFZ. An alternative explanation for the existence of fraction H is that *TbPIF5* overexpression somehow causes failure of the coordination of leading and lagging strand replication, so that the lagging strand is now synthesized continuously. Further studies are needed to test this possibility.

Here we found that TbPIF5 plays an important role in minicircle Okazaki fragment maturation. Our most significant finding was that overexpression of *TbPIF5* causes accumulation of fraction H, which is a minicircle species that contains a growing lagging strand (ranging from 0.1 kb to 1 kb) on the 1 kb L-strand templates. We now propose a model explaining how *TbPIF5* overexpression causes accumulation of fraction H (Fig. 7B). As discussed above (and diagramed in Fig. 7A), Okazaki fragment joining in wild type cells does not occur until after minicircle progeny have segregated and migrated to the antipodal sites. TbPIF5 (alone or together with other proteins) likely unwinds RNA primers, generating flaps that are subsequently degraded. The gaps are filled and repaired probably by DNA polymerase β and DNA ligase kβ. To prevent pre-maturation of Okazaki fragments, cells must tightly control the recruitment of some key enzymes such as TbPIF5. For example, TbPIF5 may bind to the minicircle progeny only after their segregation and migration to the antipodal sites. It would not be surprising that overexpression of *TbPIF5* perturbs the timing and location of Okazaki fragment processing. Excess TbPIF5 could bind to minicircle θ-structures, triggering premature removal of primers (Fig. 7B) and permitting joining of Okazaki fragments. If TbPIF5 also removes RNA primers that are not yet extended by a DNA polymerase, then further extension of the H-stand would be effectively blocked. L-strand synthesis would proceed to completion, allowing segregation of a sister with a full length newly-synthesized L-strand and another with a truncated H strand in which the Okazaki fragments had been joined. The latter molecules, with a heterogeneously-sized H-strand, form fraction H. Topoisomerase II might not recognize these molecules and therefore fail to reattach them to the network. Thus, fraction H gradually accumulates, presumably within the antipodal sites. This defect in minicircle attachment could explain the shrinking and eventual loss of kDNA that occurs following overexpression of *TbPIF5*. Further studies are needed on this helicase and other proteins involved in primer removal to fully understand the mechanism of minicircle Okazaki fragment processing.

## Materials and Methods

### Trypanosomes, transfections

Procyclic strain 29-13 (from G. Cross, Rockefeller University) was used for RNAi. Procyclic strain 927 was used for the localization experiment. Conditions for cell culture and transfection were described previously [18],[46].

### RNAi

The first 500 bp of the *TbPIF5* coding sequence were PCR-amplified using genomic DNA isolated from procyclic strain 427 and inserted into the pJZM and stem-loop vectors [18]. RNAi methodologies were described previously [18].

### Other methods

DNA and RNA purification, gel electrophoresis, Southern blotting, Northern blotting, and sucrose gradient sedimentation were performed as described previously [20]. The *TbPIF5* knockout was conducted as described previously [47]. Electron microscopy of isolated kDNA networks was done as described [48].

### *Myc*-tagging of *TbPIF5*

Fragments of the 3′-end of *TbPIF5* coding region (500 bp) and its neighboring 3′ untranslated region (500 bp) were PCR amplified using primers a–d: a, 5′GACCGGTACCCGTCTCACGCGCTTACCTATTG 3′; b, 5′ GCAGCTCGAGTTCTTCCACTTCCCCTTCATACTCCCC 3′; c, 5′ GCGGGGATCCCCGAGAGCGATGAGCGAAAAAG 3′; d, 5′ GCATCGGGGCGGCCGCACTCTCTCTCTCTCCATCTATGAATGC 3′. PCR products were inserted into pMOTag33M [49]. After digestion with Acc65I and NotI, the DNA fragments were transfected into procyclic strain 927.

### Protein expression and purification

The coding sequence (minus the first 49 amino acids which constitute a predicted mitochondrial targeting signal) was amplified by PCR, cloned into pET28a (Novagen), and transformed into the *E. coli* Rosetta™ (DE3) pLysS strain (Novagen). The cells were inoculated into 500 ml of LB medium (containing 34 µg/ml chloramphenicol and 30 µg/ml kanamycin) and grown at 37°C to an OD_600 nm_ of 0.6. After addition of 1 mM IPTG, the culture was incubated for another 3 h at 25°C. Cells were harvested by centrifugation (8000 g, 10 min) and the cell pellet was resuspended in 20 ml buffer A (50 mM sodium phosphate, 300 mM NaCl, 10 mM imidazole, pH 8.0). After lysis by sonication, the suspension was centrifuged (10000 g, 30 min) and the supernatant was mixed gently with 2 ml Ni-NTA slurry (Qiagen) (1 h, 4°C). The Ni-NTA beads were then washed 4 times with 2 ml buffer B (50 mM sodium phosphate, 300 mM NaCl, 20 mM imidazole, pH 8.0). Proteins were eluted 3 times with 0.5 ml buffer C (50 mM sodium phosphate, 300 mM NaCl, 250 mM imidazole, pH 8.0). The eluates were dialyzed overnight at 4°C against buffer D (25 mM Tris-HCl, 300 mM NaCl, 1 mM DTT, pH 7.5). The samples were loaded onto a 0.5 ml heparin-Sepharose FF (Bioscience Healthcare) column equilibrated with the same buffer. Recombinant protein was eluted at 0.8 M NaCl and dialyzed against buffer E (25 mM Tris-HCl, 100 mM NaCl, 1 mM DTT, pH 7.5). Recombinant TbPIF5 is very unstable and it was freshly prepared for the activity assays.

### Enzymatic assays of recombinant TbPIF5

For ATPase assay, recombinant TbPIF5 (10, 20, and 50 ng) was incubated (20 µl reaction, 10 min, 37°C) with 8.25 nM [γ-^32^P] ATP (6000 Ci/mmol), 150 µM non-radioactive ATP, 50 mM Tris-HCl, pH 8.5, 50 mM NaCl, 2 mM DTT, 2 mM MgCl_2_, 0.25 mg/ml bovine serum albumin, and 50 ng M13mp18 ssDNA. Samples (1 µl) were spotted onto a polyethyleneimine-cellulose plate (J. T. Baker, USA) and developed in 1.0 M formic acid/0.5 M LiCl followed by autoradiography. For helicase assays, the M13-based substrate was constructed as described [50] and the substrates for polarity assay were made as described [51]. Assays (20 µl each) contained various amounts of TbPIF5, 50 mM Tris-HCl, pH 8.5, 50 mM NaCl, 2 mM DTT, 2 mM MgCl_2_, 2 mM ATP, 0.25 mg/ml bovine serum albumin, and the substrate (15 fmol). Reactions were incubated at 37°C for 10 min and subjected to electrophoresis with a 12% polyacrylamide gel in 0.5×TBE (150 V, 1 h). The gel was dried and autoradiographed.

## Supporting Information

Figure S1 *TbPIF5* RNAi and knockout. (A) Effect of *TbPIF5* RNAi on cell growth. RNAi was induced at day 0. The value of parasites/ml on the y-axis is the measured value times the dilution factor. Inset, Northern blot showing level of *TbPIF5* mRNA (∼3.5 kb) without or with RNAi. The same blot was probed for the hexose transporter gene which provided the load control. (B) Southern blot analysis of DNA from cells in which one allele of *TbPIF5* had been knocked out. After digestion with the indicated restriction enzymes, total cellular DNA (1×10^6^ cell equivalents/lane) was fractionated on a 1% agarose gel. Southern blots were probed for *TbPIF5* gene. The diagram shows the restriction enzyme sites surrounding the *TbPIF5* gene locus. +1 represents the start site of the *TbPIF5* coding sequence. Other numbers marking restriction sites in diagram or fragment sizes in blot were determined from the genomic sequences (www.genedb.org). The positions of the nearest NheI and KpnI sites upstream of *TbPIF5* gene are −58179 and −15225, respectively. These sites are not shown in the diagram and the upstream fragment is too large to be resolved by this gel. *TbPIF1* gene is used as a loading control (Load).
(0.15 MB PDF)
