# Supplementary figures and images for: TbPIF5 Is a Trypanosoma brucei Mitochondrial DNA Helicase Involved in Processing of Minicircle Okazaki Fragments
Source: PLoS Pathog. 2009 Sep 25;5(9):e1000589. doi: 10.1371/journal.ppat.1000589 (PMC2743194; doi:10.1371/journal.ppat.1000589)

**A**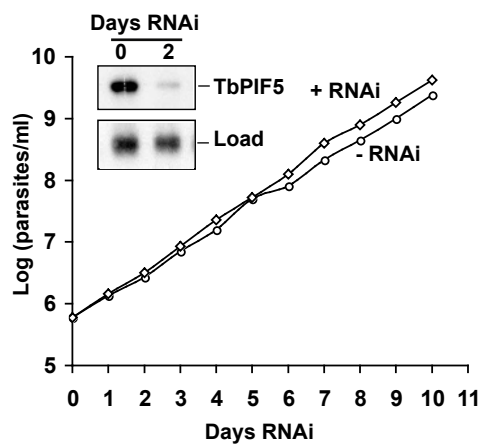**B**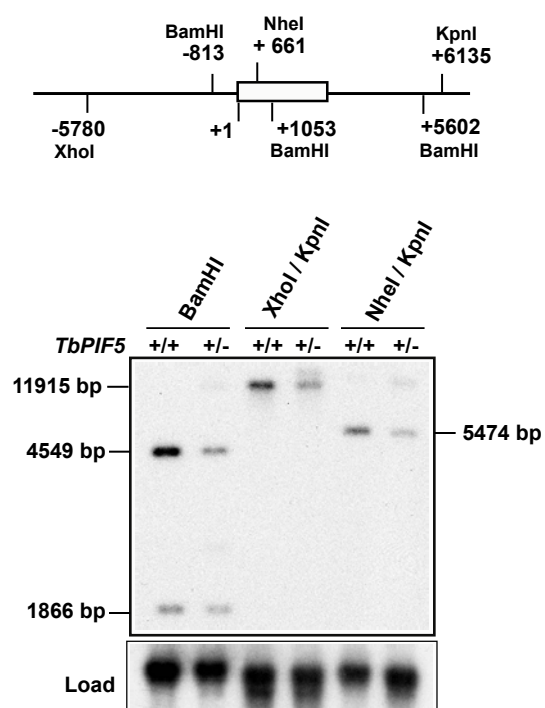

Supplement: Figure S1 — TbPIF5 RNAi and knockout. (A) Effect of TbPIF5 RNAi on cell growth. RNAi was induced at day 0. The value of parasites/ml on the y-axis is the measured value times the dilution factor. Inset, Northern blot showing level of TbPIF5 mRNA (∼3.5 kb) without or with RNAi. The same blot was probed for the hexose transporter gene which provided the load control. (B) Southern blot analysis of DNA from cells in which one allele of TbPIF5 had been knocked out. After digestion with the indicated restriction enzymes, total cellular DNA (1×10^6^ cell equivalents/lane) was fractionated on a 1% agarose gel. Southern blots were probed for TbPIF5 gene. The diagram shows the restriction enzyme sites surrounding the TbPIF5 gene locus. +1 represents the start site of the TbPIF5 coding sequence. Other numbers marking restriction sites in diagram or fragment sizes in blot were determined from the genomic sequences (www.genedb.org). The positions of the nearest NheI and KpnI sites upstream of TbPIF5 gene are −58179 and −15225, respectively. These sites are not shown in the diagram and the upstream fragment is too large to be resolved by this gel. TbPIF1 gene is used as a loading control (Load). (0.15 MB PDF) [file ppat.1000589.s001.pdf]
